# Viral pathogen detection in U.S. game-farm mallard (*Anas platyrhynchos*) flags spillover risk to wild birds

**DOI:** 10.3389/fvets.2024.1396552

**Published:** 2024-05-27

**Authors:** Brian P. Bourke, Robert J. Dusek, Koray Ergunay, Yvonne-Marie Linton, Sergei V. Drovetski

**Affiliations:** ^1^Walter Reed Biosystematics Unit, Museum Support Center MRC-534, Smithsonian Institution, Suitland, MD, United States; ^2^One Health Branch, Walter Reed Army Institute of Research, Silver Spring, MD, United States; ^3^Department of Entomology, Smithsonian Institution—National Museum of Natural History, Washington, DC, United States; ^4^U.S. Geological Survey, National Wildlife Health Center, Madison, WI, United States; ^5^Hacettepe University, Department of Medical Microbiology, Ankara, Türkiye; ^6^U.S. Geological Survey, Eastern Ecological Science Center at the Patuxent Research Refuge, Laurel, MD, United States

**Keywords:** Mallard, pathogen, virus, game-farm, spillover

## Abstract

The threat posed by emerging infectious diseases is a major concern for global public health, animal health and food security, and the role of birds in transmission is increasingly under scrutiny. Each year, millions of mass-reared game-farm birds are released into the wild, presenting a unique and a poorly understood risk to wild and susceptible bird populations, and to human health. In particular, the shedding of enteric pathogens through excrement into bodies of water at shared migratory stop-over sites, and breeding and wintering grounds, could facilitate multi-species long-distance pathogen dispersal and infection of high numbers of naive endemic birds annually. The Mallard (*Anas platyrhynchos*) is the most abundant of all duck species, migratory across much of its range, and an important game species for pen-rearing and release. Major recent population declines along the US Atlantic coast has been attributed to game-farm and wild mallard interbreeding and the introduction maladaptive traits into wild populations. However, pathogen transmission and zoonosis among game-farms Mallard may also impact these populations, as well as wildlife and human health. Here, we screened 16 game-farm Mallard from Wisconsin, United States, for enteric viral pathogens using metatranscriptomic data. Four families of viral pathogens were identified – *Picobirnaviridae* (Genogroup I), *Caliciviridae* (Duck *Nacovirus*), *Picornaviridae* (Duck *Aalivirus*) and *Sedoreoviridae* (Duck *Rotavirus* G). To our knowledge, this is the first report of *Aalivirus* in the Americas, and the first report of *Calicivirus* outside domestic chicken and turkey flocks in the United States. Our findings highlight the risk of viral pathogen spillover from peri-domestically reared game birds to naive wild bird populations.

## Introduction

Emerging infectious diseases are a major threat to global public and animal health (1). Since the beginning of the 21st century, there have been a series of severe global disease outbreaks primarily caused by zoonotic viral pathogens (2–5). Investigations into the zoonotic origins of recent outbreaks have flagged the risks posed by birds, most notably in the transmission of West Nile Virus (WNV) (6, 7) and Highly Pathogenic Avian Influenza (HPAI) (8, 9).

Each year, billions of birds undertake seasonal migration, with some moving between the high latitudes of the northern and southern hemispheres and travelling tens of thousands of kilometers annually. Such vagility provides bird pathogens a unique opportunity to be rapidly dispersed across the globe (10). Migratory birds often congregate at stopover sites, which offer shelter to rest and feed, and the high bird densities and multi-species clustering increases opportunities for pathogen transmission (11, 12). The migratory routes of birds have played a key role in the global transmission of HPAI (13). The arrival of HPAI in North America in 2014, and 2022, is attributed to birds using migratory flyways on their way from summer breeding grounds in Siberia (14) and northwest Europe (15), respectively. The transmission of this virus occurs via the fecal-oral route and typically through inhalation of infectious aerosols, or contact with contaminated water (16, 17). Predatory and scavenging birds can also become infected by consumption of infected prey and/or carcasses (18, 19). In addition to birds serving as highly mobile pathogen reservoirs, they can transport ectoparasites, which may in turn serve as vectors of pathogens. Birds can thus also be vehicles for the transport of vector-borne pathogens along migratory flyways. Studies from Europe and North America indicate migratory birds carry exotic ectoparasites, including ticks (20, 21). Studies screening these hitch-hiking ticks have confirmed Crimean-Congo hemorrhagic fever (CCHF) virus (22–25), and *Borrelia* (26) and *Babesia* (27) species that can cause disease in many vertebrates, including humans.

In Europe and North America, game birds (e.g., Common pheasant [*Phasianus colchicus*], Quail [*Colinus virginianus*], Chukar partridge [*Alectoris chukar*], red-legged partridges [*Alectoris rufa*], Mallard [*Anas platyrhynchos*]) are reared *en mass* and released into the wild to bolster game populations for hunting (28–30). The scale of this industry is vast, involving many tens of millions of birds annually. In Europe, game-farm Mallard releases are estimated at almost 3 million annually and are comparable in size to the continent’s wild mallard breeding population (30–33). In North America more than 300 licensed hunting preserves, located primarily along the Atlantic and Mississippi flyways, release almost 300,000 game-farm Mallard annually (34). Interbreeding between game-farm and wild Mallard populations is believed to have formed a Mallard hybrid swarm in North America, resulting in population decline due to the introduction of artificially created and maladaptive genes (35, 36). However, interactions among game-farm and wild Mallard can also introduce emerging and novel pathogens into naive wild bird populations, which may also explain population declines. While pathogen transmission risks associated with wild and commercial poultry flocks has been well documented (37–39), the transmission risk from game-farm birds to wild birds, and other vertebrate hosts, including humans, is poorly understood (40).

The global scale and magnitude of avian viral outbreaks has the potential not only to threaten vulnerable species with extinction (41–43) and decimate wild and domestic bird populations (44–46) across the globe, but is also known to spill over into terrestrial and marine mammal species, offering opportunities for adaptation, new transmission cycles, and zoonosis (47). Characterizing viral pathogen diversity particularly at avian, human, and environment interfaces is therefore a key element to safeguarding global health security. Although the risks and impacts of HPAI and WNV have been widely reported, a variety of other, less well-characterized viral pathogens also pose a threat to bird populations, the food industry, and human health. These include Newcastle Disease Virus (NDV), which causes disease in domestic and wild birds (48), and Rotavirus (ROV) and Eastern equine encephalitis virus (EEEV), which cause disease in both birds (49, 50) and humans (51, 52).

Advances in whole genome sequencing technologies allow new opportunities to screen, identify, and characterize viral pathogens. Metagenomics and metatranscriptomics allows the full nucleotide content of a sample to be sequenced concurrently, thus providing an agnostic approach to characterizing the viral community. Here we use a metatranscriptomics approach to analyze the viromes of game-farm Mallard to expose known and novel pathogens that may pose a risk to wild mallard and susceptible wild bird populations and offer a potential route for reverse zoonosis in wildlife.

## Materials and methods

### Sampling and nucleic acid extraction

We sampled the cecal content of 16 fully grown 8-week-old (juvenile) female Mallard that were raised in an open-air pond in Wisconsin, United States. None of the birds showed signs of ill health. Approximately 100 mg of cecal content was sampled from each bird immediately after euthanasia by CO_2_ according to the protocol approved by the ACUC of the U.S. Geological Survey, National Wildlife Health Center (tracking number EP210811). The cecum wall was punctured with a sterile scalpel blade and cecal content (two pin-head size drops) was pushed directly into a 2 mL screw cap tube pre-filled with 1 mL of DNA/RNA Shield and a mix of 0.5 mm and 0.1 mm ultra-high density BashingBeads (Zymo Research, Irvine, CA, United States). Samples were stored at 4°C until nucleic acid extraction within 30 days of collection. Total RNA from the cecal samples was extracted using the ZymoBIOMICS DNA/RNA Miniprep Kit (Zymo Research), according to the manufacturer’s instructions. The RNA concentration of each sample was then measured on a Qubit 4.0 (ThermoFisher Scientific, Waltham, MA, United States) using the High-Sensitivity RNA Assay Kit (ThermoFisher Scientific).

### Library preparation and sequencing

Samples were prepared for sequencing by depleting ribosomal RNA using QIAseq FastSelect -rRNA HMR Kit (QIAGEN, Germantown MD) and RNA libraries were then prepared using the NEB Ultra II RNA kit (New England Biolabs, Inc., Ipswich, MA, United States). All libraries were sequenced simultaneously on an Illumina HiSeq 4,000 platform (2 × 150 bp; Illumina, Inc., San Diego, CA, United States). Library preparation and sequencing was performed by GeneWiz/Azenta Life Sciences (South Plainfield, NJ, United States). The number of pair-ended reads obtained from individual samples ranged from 49,218,452 to 74,399,926 [median 60,564,664].

### Data analysis

Demultiplexed raw Illumina data were first adaptor trimmed and quality filtered using fastp v0.23.3 (−-qualified_quality_phred = 15; −-unqualified_percent_limit = 40) (53, 54). Following this step, host genome reads were removed from the dataset using KneadData v0.12.0^1^ and the Mallard reference assembly.^2^ The bowtie alignment option within KneadData was set to “--very-sensitive-local” (i.e., -D 20; -R 3; -N 0; -L 20; -i S,1,0.50).

The cleaned data was then *de-novo* assembled using MEGAHIT v1.2.9 and its “basic usage” setting for paired end libraries (55). *De novo* assembled contigs were then aligned to the National Center for Biotechnology Information (NCBI) protein non-redundant (nr) database (http://ftp.ncbi.nlm.nih.gov/blast/db/FASTA/nr.gz; accessed April 5 2023) using Diamond (−-long-reads; −-evalue 1e-9) (56, 57) and taxonomically binned using Megan v6.24.20 (−-minSupport 1; −-minPercentIdentity 70; −-maxExpected 1.0E-9; −-lcaAlgorithm longReads; −-lcaCoveragePercent 51; −-longReads) (58, 59). Contigs classified as viruses of potential pathogenic importance (vertebrate pathogens) based on a review of the literature were then exported from Megan. Putative Open Reading Frames (ORFs) and amino acid sequences were predicted using ORF finder.^3^ ORFs were then aligned with close and/or congeneric relatives, as described in the International Committee on Taxonomy of Viruses (ICTV) report chapters,^4^ using Muscle implemented in SeaView v5.0.4 (60). The removal of spurious and/or poorly aligned regions from the multiple sequence alignment was achieved using trimAl v1.4 (61) and its automated heuristic approach (option-automated1).

Maximum likelihood phylogenetic analysis was performed in IQ-TREE 2 v2.2.0 (62) with 1,000 replicates. Optimal model selection was performed using the -m MFP option, and the models were restricted to those designed for viruses using the -msub viral option. The resulting consensus trees contain UFBoot support values, which are more unbiased than standard nonparametric bootstrap values, and support for a clade considered at ≥95%.

Screening for recombination among virus genomes was performed through algorithms implemented in the RDP4 software using the seven primary algorithms available (RDP, GENECONV, BOOTSCAN/RECSCAN, MAXCHI, Chimaera, 3SEQ and SISCAN) (63).

## Results

A variety of viruses were detected in the metatranscriptomic data from these Mallard gut samples (Table 1). None of the viruses detected displayed a signal of recombination in a majority of the seven default RDP4 tests.

**Table 1:** Summary of metatranscriptomic data and viral pathogen detection from the cecum of 16 captive Mallard from Wisconsin, United States (Bioproject: PRJNA1045981).

Sample ID	SRA Acc.	Pathogen [Genbank Acc.]
PI202111	SRR26965775	None
PI202112	SRR26965774	Duck Calicivirus (Nacovirus) [PP472417]
		Duck Rotavirus G [PP558645]
PI202113	SRR26965767	None
PI202114	SRR26965766	Duck Aalivirus A [PP590634]
PI202115	SRR26965765	Picobirnavirus (Genogroup I) [OR820937, PP512782]
PI202116	SRR26965764	Duck Calicivirus (Nacovirus) [OR837086, PP512780, PP512781]
PI202117	SRR26965763	None
PI202118	SRR26965762	None
PI202119	SRR26965761	Duck Aalivirus A [OR769080]
PI202120	SRR26965760	None
PI202121	SRR26965773	Duck Aalivirus A [OR769079]
PI202122	SRR26965772	None
PI202123	SRR26965771	None
PI202124	SRR26965770	Duck Rotavirus G [OR820938, PP558642, PP558644, PP558646, PP590633]
PI202125	SRR26965769	None
PI202126	SRR26965768	Duck Rotavirus G [PP558643]

### Picobirnaviruses

Two *de novo* assembled contigs from sample PI202115 were classified as Picobirnavirus in Diamond-Megan analysis. They were from the two linear dsRNA Picobirnavirus segments (dsRNA1 and dsRNA2). The contig from the dsRNA1 segment (1,375 base pairs) represented the viral capsid protein (ORF3), which was 457 amino acids in length. Although the phylogenetic signal from the viral capsid protein was poor, based on the lack of branch support (Supplementary Figure S1), the PI202115 sequence clustered (UFBoot support: 100%) with chicken picobirnavirus from Brazil (Genbank Acc. AXL64599), at 91.98% identity.

**Figure 1:**
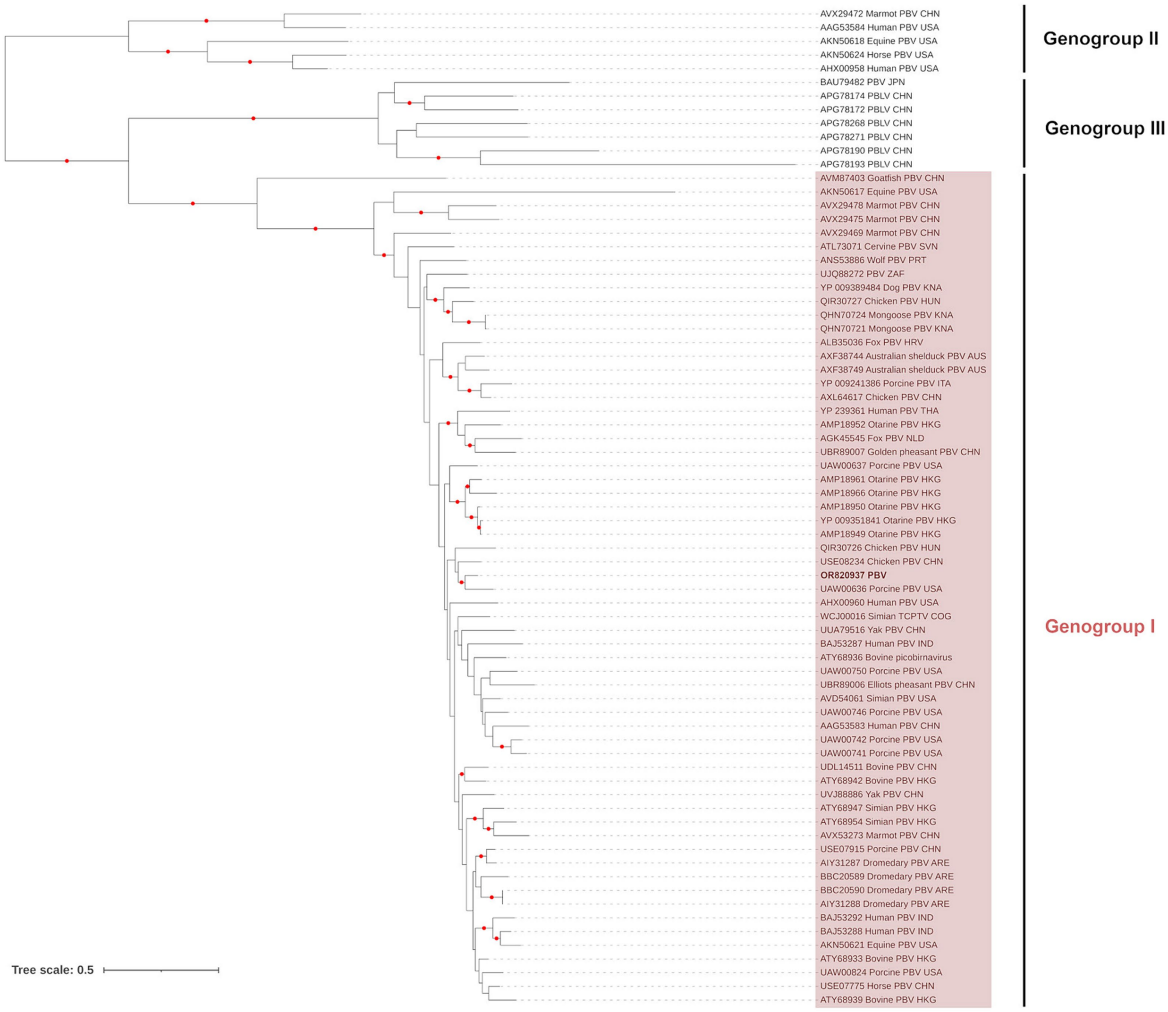
Maximum likelihood (extended majority-rule consensus) tree of the family *Picobirnaviridae* using the RdRP region. Red circles indicate branches with UFBoot support greater than 95%. Picobirnavirus from sample PI202115 (Genbank Acc. OR820937) is highlighted in boldface.

The contig from the dsRNA2 segment (1,669 base pairs; Genbank Acc. OR820937) represented the viral RNA-dependent RNA polymerase (RdRp) and was 529 amino acids in length. The sequence contains the seven conserved core polymerase domain motifs of picobirnaviruses (64). Phylogenetic analysis of the RdRP region showed that the PI202115 sequence clustered (UFBoot support: 95%) with a pig picobirnavirus (UAW00636), within the Genogroup I clade (UFBoot support: 100%), which can be resolved from the Genogroup II (UFBoot support: 100%) and Genogroup III (UFBoot support: 100%) found in the *Picobirnaviridae* tree (Figure 1), as described in the ICTV Report on *Picobirnaviridae* (65). The picobinavirus RdRp region from PI202115 shared 80.64% identity (based on amino acid sequence) with the pig picobirnavirus (UAW00636).

### Caliciviruses

Taxonomic binning of the *de novo* assembled contigs found one (2,560 bp) and six (1,020–5,850 bp) contigs with Calicivirus (CV) and descendant taxa classifications from samples PI202112 and PI202116, respectively. Although the contigs did not represent the complete genome, the genome’s two open reading frames (ORF1 and ORF2) were detected in sample PI202116, whereas only ORF1 was identified from sample PI202112. Although coverage was complete for ORF2, individual contig coverage did not exceed 70% for ORF1.

**Figure 2:**
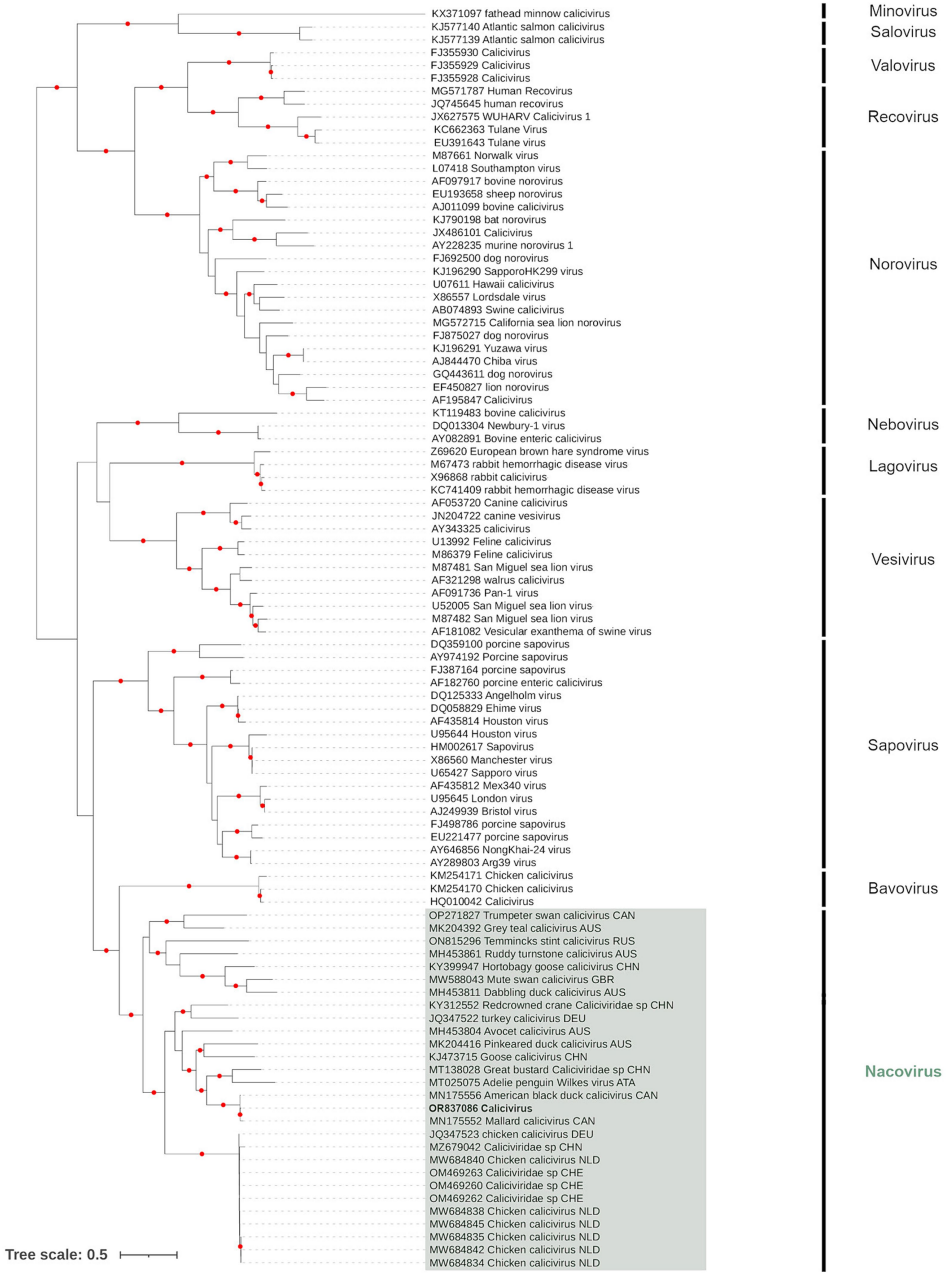
Maximum likelihood (extended majority-rule consensus) tree of the family *Caliciviridae* using the VP1 region. Red circles indicated branches are supported at greater than 95% (UFBoot support). Calicivirus from sample PI202116 (Genbank Acc. OR837086) is highlighted in boldface.

Only a single contig, from PI202116 (Genbank Acc. OR837086), overlapped with the complete major capsid protein (VP1) amino acid sequence of ORF1, which was the region recommended for CV genus demarcation and species resolution by the ICTV. Phylogenetic analysis of VP1 showed the PI202116 contig belonged to the Nacovirus group of CVs, which are typically associated with avian species (Figure 2). The contig was a sister (UFBoot support: 100%) to the nacovirus detected in a Mallard from Canada (Genbank Acc. MN175552), with which it shared 95.58% identity (based on amino acid sequence). This sister pair formed a clade (UFBoot support: 100%) with an additional nacovirus detected in an American black duck, *Anas rubripes*, from Canada (Genbank Acc. MN175556).

When the full ORF1 region was analyzed, the contigs from PI202112 and PI202116 overlapping this region were found within the nacovirus group, but within distinct clades. Although nacovirus from PI202116 remained within a clade formed by the nacovirus from Mallard and American black duck, the nacovirus from PI202112 is most closely associated with one identified in a “Dabbling Duck” from Australia (Genbank Acc. AXF38657), at 87.37% amino acid base identity (Supplementary Figure S2). These two sequences formed a sister relationship with a Hortobagy goose calicivirus (Genbank Acc. ARM65436), which has previously been proposed as a new CV genus, denoted “*Sanovirus*” (66). Analysis of the minor structural protein (VP2 located in ORF2) found little phylogenetic signal with a poorly supported tree.

### Aaliviruses

Taxonomic binning of the *de-novo* assemblies resulted in one (1,073 bp), five (1,195–3,105 bp) and three (1,229–3,198 bp) contigs with *Aalivirus* (Pacific black duck [*Anas superciliosa*] *Aalivirus* A/*Aalivirus* A) consensus classifications for samples PI202114, PI202119 and PI202121, respectively. The genome of *Aalivirus* is organized into viral structural proteins (P1) and non-structural proteins (P2 and P3). Contigs from all three proteins were detected in the data, with P1 found in sample PI202114 and PI202121, and P2 and P3 found in PI202119 and PI202121. All four canonical cleavage sites (DxExNPG|P) described for the P2 protein (2A region) in *Aalivirus* (67) were detected in contigs. Characteristic motifs conserved in picornaviruses were detected in helicase, from the P2 protein (GEPGSGKS and DDLGQ), and in cysteine protease (GSCG) and RdRp (KDELR, DFKKFD, GGMCSGSPCTTVLNNT and FLKR) from the P3 protein.

**Figure 3:**
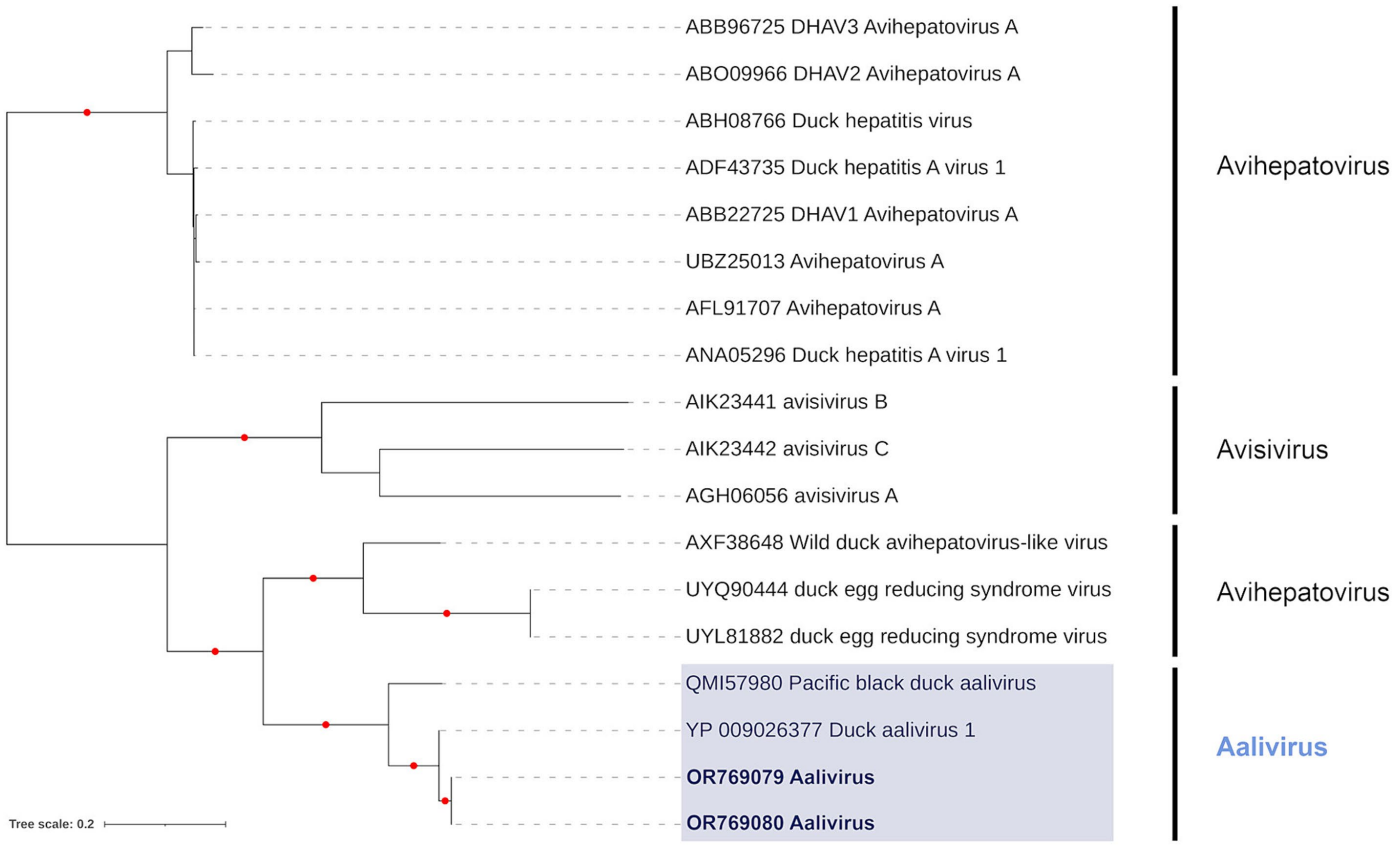
Maximum likelihood (extended majority-rule consensus) tree of the Genus *Aalivirus* and its closest relatives using the RdRp region. Red circles indicated branches are supported at greater than 95% (UFBoot support). Aaliviruses from samples PI202119 (Genbank Acc. OR769080) and PI202121 (Genbank Acc. OR769079) are highlighted in bold.

Phylogenetic analysis of the RdRp region in P3 showed that contigs from samples PI202119 (Genbank Acc. OR769080) and PI202121 (Genbank Acc. OR769079) clustered together (Figure 3), and these formed a sister relationship with the Duck aalivirus A found in a domestic Mallard from China (Genbank Acc. YP_009026377). These PI202119 and PI202121 contigs had percent identities (based on amino acid sequence) of 98.99 and 98.78% with Duck aalivirus A (Genbank Acc. YP_009026377), respectively. Phylogenetic analysis of the *Aalivirus* from sample PI202114 using a P1 region sequence alignment (not shown) placed it as a sister to Duck aalivirus A from Australia (Genbank Acc. QMI57977). Analysis of helicase and peptidase C3, regions previously used for the phylogenetic analysis of the aalivirus group (67), found little phylogenetic signal, with poorly supported trees.

### *Rotavirus* G

Taxonomic binning of contigs resulted in two (1,266–1,589 bp), seven (1,022–3,507 bp) and one (1,345 bp) contigs with *Rotavirus* G (*Rotavirus* G and Pacific black duck *Rotavirus* G) consensus classifications for samples PI202112, PI202124, and PI202126, respectively. The *Rotavirus* G genome is organized into 11 linear dsRNA segments and contigs from seven of these segments (Segments 1 to 7) were detected among the samples analyzed. Three conserved VP1 protein motifs characteristic of rotaviruses (ANKIILYTDVSQWDAS, LKIRYHGVASGEKTTKIGNSYANVALI, and LRVDGDDNVIT) (68, 69) were identified from segment 1 in sample PI202124. An additional conserved rotavirus motif from the VP3 protein in segment 3 (ALYSISN) was also found in sample PI202124.

**Figure 4:**
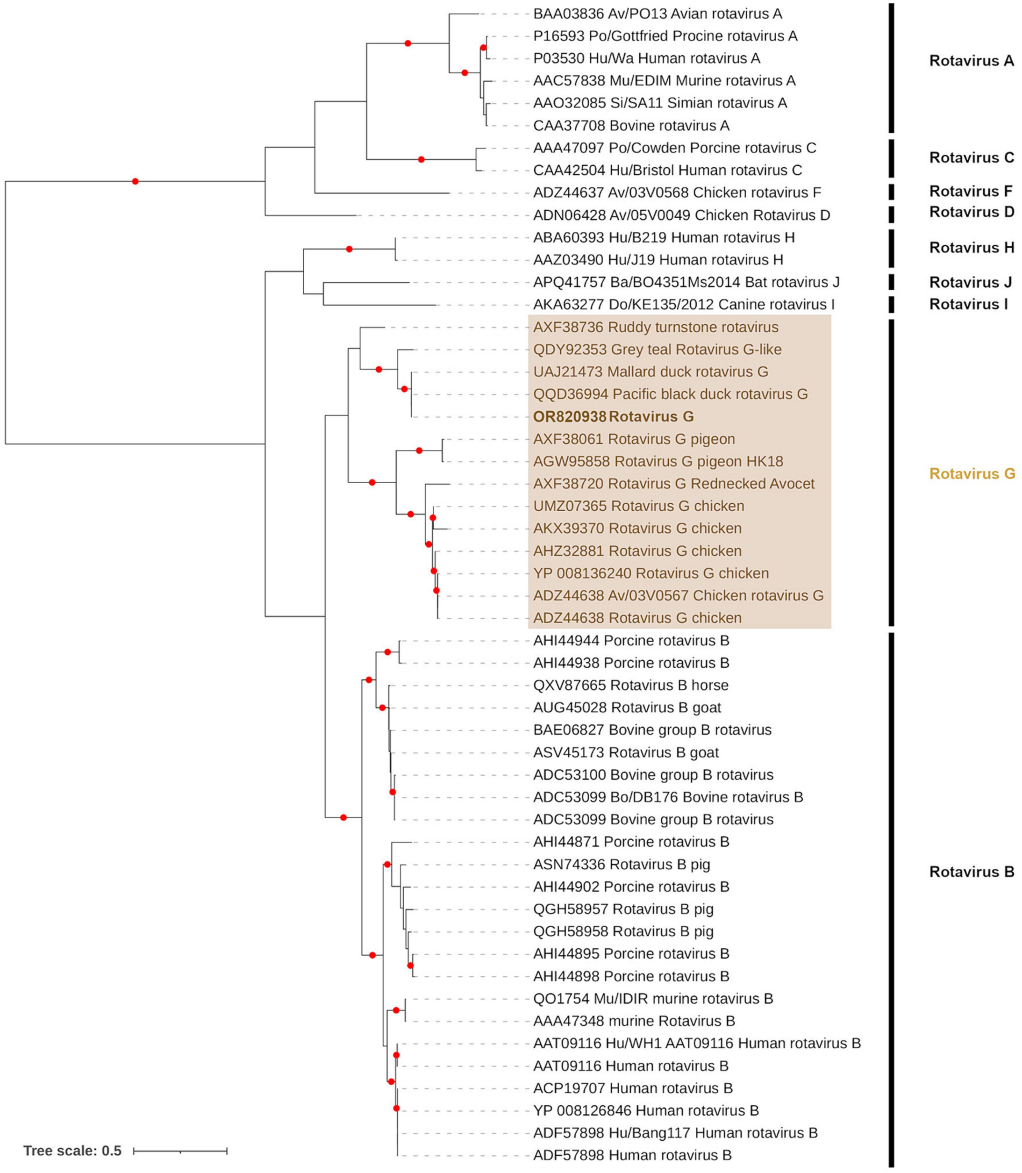
Maximum likelihood (extended majority-rule consensus) tree of the Genus *Rotavirus* using the VP6 region. Red circles indicated branches are supported at greater than 95% (UFBoot support). Calicivirus from sample PI202124 (Genbank Acc. OR820938) is highlighted in bold.

In accordance with the criteria for genus demarcation and species demarcation by the ICTV, we aligned our data with all known species of *Rotavirus* (*Rotavirus* A–D, F–J) at the inner capsid protein (VP6) region of segment 6. Only sample PI202124 had *Rotavirus* G contigs overlapping this region. The *Rotavirus* G from PI202124 (Genbank Acc. OR820938) was most closely related to Pacific black duck *Rotavirus* G (Genbank Acc. QQD36994) and Mallard *Rotavirus* G (Genbank Acc. UAJ21473) identified from Australia (Figure 4) with 100 and 99.75% identity (based on amino acid sequence), respectively.

Although phylogenetic branch support was poor in Segments 5 (Sample PI202124) and 7 (Samples PI202112 and PI202124) contigs, the remaining Segments 1 to 4 contigs from samples PI202112, PI202124, and PI202126 were placed in sister positions with either Pacific black duck *Rotavirus* G or Mallard *Rotavirus* G from Australia (Supplementary Figure S3).

## Discussion

Birds are among the most vagile of vertebrates and have the capacity to transport a range of viral pathogens that pose a risk to other wild birds, commercial poultry, wildlife, domestic animals, and human health. Viral pathogens that can be transmitted through enteric routes are of considerable risk to commercial poultry flocks and global food supply chains, and the potential risks of zoonosis, most notably in relation to HPAI in recent years, are of major public health concern (70). Characterizing the viral diversity present in migratory birds is thus an essential component of a viral pathogen surveillance, diagnostic, and early warning system (10). Here we have characterized viral pathogens present in the enteric system (cecum) of a group of Mallard captive-raised for release during hunting season. Mallard is the most abundant of all duck species and one that is highly migratory across much of its range with a potential to move between continents (71). We detected four families of viral pathogens in our dataset - *Picobirnaviridae* (Genogroup I), *Caliciviridae* (duck *Nacovirus*), *Picornaviridae* (duck *Aalivirus*) and *Sedoreoviridae* (duck *Rotavirus* G).

The first group of viruses detected from our study group belong to the genus *Aalivirus* from the family *Picornaviridae*. To our knowledge, this is the first record of aalivirus in the Americas, with records from only Australia (72), China (67), and Iran (73). This genus comprises linear, positive sense, unsegmented ssRNA genomes (~9,000 bp), which are made up of a polyprotein divided into three functional regions, P1, P2, and P3. The first aalivirus was detected in diseased domestic Mallard from China in 2014 (67). These were found to be most closely related to duck hepatitis A virus from the genus *Avihepatovirus*, which causes high mortality among young ducks and is of considerable importance to the poultry industry (74). Work on *Aalivirus* since its discovery has failed to establish effective culturing methods, and the epidemiology of aalivirus remains largely unknown. In the current study, the aaliviruses detected were most closely related to the original duck aalivirus first described in China (67) and one subsequently identified from Australia (75). Further agnostic sequencing of the enteric systems of potential host species across broad geographical ranges could substantially contribute to novel *Aalivirus* discovery, characterizing phylogenomic relationships, and better understanding this viruses phylodynamics.

The second group detected is from the family *Caliciviridae*. This is a family of viruses comprising linear, positive sense, unsegmented ssRNA genomes (6,400–8,500 bp), and consist of a major capsid protein, VP1, a minor structural protein, VP2, and VPg. Caliciviruses have been detected in a wide variety of animals, where they are associated with veterinary diseases, such as gastroenteritis, respiratory illnesses, and hemorrhagic fever (76–79). They are believed to be spread primarily by the fecal-oral route (80). In humans, they are among the most important causes of non-bacterial gastroenteritis outbreaks and epidemics (81–83). They are routinely detected in birds but, without reliable virus isolation, their epidemiology remains poorly known. The caliciviruses detected in our study group belonged to the genus *Nacovirus*, which is one of the 11 recognized genera within the family *Caliciviridae*, and whose genome is comprised of two open reading frames (ORF1 And ORF2). The nacovirus that was detected in PI202116 and could be characterized according to ICTV criteria (the VP1 amino acid sequence of ORF1) was found to be closely related to a duck nacovirus detected in a Mallard from Canada (84). Although calicivirus has been detected in a variety of wild waterfowl in Canada (84, 85) and from domestic poultry in the United States (86), to our knowledge this is the first time this virus has been reported outside domestic chicken and turkey flocks in the United States. Further characterization of calicivirus diversity extant in wild bird populations from North America could enhance our understanding of the potential for transmission between wild, captive reared and domestic birds.

The third group of viruses detected belong to the family *Picobirnaviridae*. This family of viruses comprises bi-segmented dsRNA genomes (4,100–4,600 bp total length). The longer of the two segments, dsRNA1, comprises three open reading frames, whereas the smaller segment, dsRNA2, encodes a viral RNA-dependent RNA polymerase (RdRp). Picobirnaviruses (PBVs) are widespread in animal gastrointestinal tracts and feces but the potential link between PBV and gastroenteritis in mammals remains controversial (87). Although information is scant, there is some evidence for inter-species transmission and zoonoses (88–90). Currently, only three species have been delimited by the ICTV – Genogroups I, II and III - based on host specificity and the strong sequence divergence of their capsid proteins. The PBV detected in the current study (found in sample PI202115) and characterized by ICTV criteria belonged to the Genogroup I. It was found to be most closely related to a Genogroup I metagenome assembled genome (MAG) detected in a pig from North Carolina, United States (91). Given the enteric nature of PBVs, there is elevated risk of transmission among birds and other animals at water bodies utilized by resident and migratory birds.

The final group of viruses detected belong to the genus *Rotavirus* (RV) from the family *Sedoreoviridae*. This genus comprises double-stranded RNA genomes with 11 segments (700 bp to 3,200 bp) contained within a non-enveloped, triple-layered capsid. These segments encompass structural proteins, including VP1, VP2, VP3, VP4, VP6, and VP7, and non-structural proteins, including NSP1, NSP2, NSP3, NSP4, NSP5, and NSP6. The genus is currently divided into nine species - rotavirus A–D and F–J – which are major causes of gastroenteritis in a wide variety of animals. In humans, RV infection is the most common cause of acute gastroenteritis worldwide, with the species responsible primarily identified as rotavirus A–C (92). The species *Rotavirus D* (93), *F,* and *G* (94) have only ever been detected in avian species. Avian rotavirus (AvRV) infection is most often encountered among young birds (1–2 weeks (50)) and prevalence of up to 85% have been found among some poultry flocks (95). Despite such high prevalence, AvRV vaccines remain unavailable. The mode of RV transmission is primarily via the fecal-oral route, and an important source of infection is contaminated water (96, 97). Although RV zoonotic risk is considered low, the frequency of reassortment among RVs may facilitate viral novelty and opportunities for major evolutionary changes that are required for successful zoonotic transmission. Such zoonotic transmission may have a variety of animal origins (98, 99). In the current study, taxonomic classification of the *de novo* assemblies revealed the presence of the bird-specific rotavirus G species. Characterization according to ICTV species demarcation found the rotavirus G species present was the same species as that found in Mallard and Pacific black duck from Australia (72). These hosts are closely related migratory species and have the potential to transport rotavirus along migratory routes and may be an important source of transmission, as has been proposed in other migratory waterfowl (100).

Each of the enteric viral pathogens detected in this study may be shed at high concentrations in feces, leading to the contamination of large water bodies utilized by waterfowl and a great variety of birds and other animals, including humans, sharing these water resources. Surface water has been shown to be a highly important medium for the transmission of viruses, including HPAI (101). The viral pathogens detected in this study are therefore potentially important candidates for water-borne disease outbreaks in wildlife, domestic animals, and humans. Although pathogen transmission between wildlife and commercial poultry is well documented (37–39), the transmission risk of pathogens from captive-bred game birds and wildlife remains poorly understood. Commercial farms each typically produce between 20,000 to 300,000 game birds annually, where individuals are initially housed in indoor brooder houses before acclimation to the outdoors after 1–2 months (28). Such high host density present can facilitate pathogen transmission and increase disease susceptibility among the captive-bred flock. The release of these flocks onto private and public lands where they mix with wild conspecifics and other wildlife may therefore represent an important source for viral transmission between wildlife, domestic animals, and humans (102, 103). It is proposed that the pervasive interbreeding of game-farm and wild Mallard in North America east (35, 104) has led to the introduction of maladaptive traits, which has been an important factor in recent population decline (35, 36). Findings from the current study propose pathogen spillover from game-farm Mallard as another possible contributing factor in recent population declines. Additionally, the Mallard is the most closely related extant species to, and possibly the common ancestor of, domestic ducks (105), thus making the interface between wildlife and domestic birds highly permeable and increasing the risks of viral spillover and spillback.

Understanding the epidemiology and etiology of game-farm pathogens first requires establishing systems of surveillance to quantify pathogen prevalence, characterize diversity, elucidate evolutionary relationships and phylodynamics and, in the absence of effective culturing approaches, proposing etiological hypotheses by investigating relationships between pathology and infection prevalence. Increasing efforts to screen game-farm flocks prior to release and redoubling efforts to characterize game-farm and wild bird viromes using agnostic sequencing approaches could help elucidate the pathogen transmission dynamics between game-farm and wild birds and reveal spillover events and may provide a means to control the release of infectious game-farm birds. In our study, despite sampling Mallard from only a single age cohort, season, and game-farm, we find a rich and important diversity of viruses. This preliminary data therefore provides a strong impetus for us to further sample game-farm and wild Mallard to test our hypothesis of spillover between game-farm and wild populations and better characterize viral diversity within these populations. Enhancing and increasing monitoring of enteric viral pathogens in water bodies near release sites, and at important stopover sites, and breeding/wintering grounds, could strengthen our ability to track emerging pathogens, identify potential hotspots and better understand their epidemiology. Agnostic sequencing approaches offer a valuable opportunity surveil a rich diversity of viruses in these water bodies, but further testing and optimization could aid in understanding the sensitivity of these sequencing approaches to presumably lower viral concentrations present in this medium. These approaches possess the means to establish a baseline for viral pathogen prevalence, diversity, and dynamics at important interfaces and among globally important bird populations and could be used to develop an early warning system for outbreaks of water-borne disease of avian origin. Such information could be utilized by One Health networks, which focus on the relationships between animal, human, and environmental health where outbreaks of emerging and neglected diseases can occur, to better develop strategies to detect, characterize and mitigate potentially pandemic pathogens.

## Data availability statement

Metatranscriptomic data generated and analyzed in this study is available from BioProject PRJNA1045981 (https://www.ncbi.nlm.nih.gov/bioproject). GenBank accessions for the viral sequences used in phylogenetic analyses are indicated in Table 1, and also available in a U.S. Geological Survey data release (106).

## Ethics statement

The animal study was approved by ACUC, U.S. Geological Survey, National Wildlife Health Center (tracking number EP210811). The study was conducted in accordance with the local legislation and institutional requirements.

## Author contributions

BB: Conceptualization, Data curation, Formal analysis, Investigation, Methodology, Visualization, Writing – original draft, Writing – review & editing. RD: Conceptualization, Resources, Writing – review & editing. KE: Formal analysis, Writing – review & editing. Y-ML: Resources, Writing – review & editing, Supervision. SD: Conceptualization, Data curation, Funding acquisition, Investigation, Resources, Writing – review & editing, Project administration, Supervision.

## Footnotes

^1^https://github.com/biobakery/kneaddata

^2^https://www.ncbi.nlm.nih.gov/datasets/genome/GCF_015476345.1/

^3^https://www.ncbi.nlm.nih.gov/orffinder

^4^https://ictv.global/report

## Author disclaimer

All or portions of the laboratory and/or data analysis were conducted in and with the support of the Laboratories of Analytical Biology (https://ror.org/05b8c0r92) of the National Museum of Natural History. The opinions or assertions contained herein are the private views of the authors, and are not to be construed as official, or as reflecting true views of the Department of the Army, Navy, or the Department of Defense. Material contained within this publication has been reviewed by the Walter Reed Army Institute of Research. There is no objection to its presentation and/or publication. Any use of trade, firm, or product names is for descriptive purposes only and does not imply endorsement by the U.S. Government.

## References

[1] Allen T Murray KA Zambrana-Torrelio C Morse SS Rondinini C Di Marco M. Global hotspots and correlates of emerging zoonotic diseases. Nat Commun. (2017) 8:1124. doi: 10.1038/s41467-017-00923-8, PMID: 29066781 PMC5654761

[2] Sullivan SJ Jacobson RM Dowdle WR Poland GA. 2009 H1N1 Influenza. Mayo Clin Proc. (2010) 85:64–76. doi: 10.4065/mcp.2009.0588, PMID: 20007905 PMC2800287

[3] Coltart CEM Lindsey B Ghinai I Johnson AM Heymann DL. The Ebola outbreak, 2013–2016: old lessons for new epidemics. Philos Trans R Soc Lond B Biol Sci. (2017) 372:20160297. doi: 10.1098/rstb.2016.0297, PMID: 28396469 PMC5394636

[4] Baud D Gubler DJ Schaub B Lanteri MC Musso D. An update on Zika virus infection. Lancet. (2017) 390:2099–109. doi: 10.1016/S0140-6736(17)31450-2 28647173

[5] Al-Awwal N Dweik F Mahdi S El-Dweik M Anderson SH. A review of SARS-CoV-2 disease (COVID-19): pandemic in our time. Pathogens. (2022) 11:368. doi: 10.3390/pathogens11030368, PMID: 35335691 PMC8951506

[6] Garmendia AE Van Kruiningen HJ French RA. The West Nile virus: its recent emergence in North America. Microbes Infect. (2001) 3:223–9. doi: 10.1016/S1286-4579(01)01374-0 11358716

[7] Ronca SE Ruff JC Murray KO. A 20-year historical review of West Nile virus since its initial emergence in North America: has West Nile virus become a neglected tropical disease? PLoS Negl Trop Dis. (2021) 15:e0009190. doi: 10.1371/journal.pntd.0009190, PMID: 33956816 PMC8101735

[8] de Jong JC Claas EC Osterhaus AD Webster RG Lim WL. A pandemic warning? Nature. (1997) 389:554. doi: 10.1038/39218, PMID: 9335492 PMC7095477

[9] Sellwood C Asgari-Jirhandeh N Salimee S. Bird flu: if or when? Planning for the next pandemic. Postgrad Med J. (2007) 83:445–50. doi: 10.1136/pgmj.2007.059253, PMID: 17621612 PMC2600097

[10] Georgopoulou I Tsiouris V. The potential role of migratory birds in the transmission of Zoonoses. Vet Ital. (2008) 44:671–7. PMID: 20411494

[11] de Angeli Dutra D Fecchio A Martins Braga É Poulin R. Migratory birds have higher prevalence and richness of avian Haemosporidian parasites than residents. Int J Parasitol. (2021) 51:877–82. doi: 10.1016/j.ijpara.2021.03.001, PMID: 33848498

[12] de Angeli Dutra D Filion A Fecchio A Braga ÉM Poulin R. Migrant birds disperse Haemosporidian parasites and affect their transmission in avian communities. Oikos. (2021) 130:979–88. doi: 10.1111/oik.08199

[13] Kilpatrick AM Chmura AA Gibbons DW Fleischer RC Marra PP Daszak P. Predicting the global spread of H5N1 avian influenza. Proc Natl Acad Sci. (2006) 103:19368–73. doi: 10.1073/pnas.0609227103, PMID: 17158217 PMC1748232

[14] Lee D-H Torchetti MK Winker K Ip HS Song C-S Swayne DE. Intercontinental spread of Asian-origin H5N8 to North America through Beringia by migratory birds. J Virol. (2015) 89:6521–4. doi: 10.1128/JVI.00728-15, PMID: 25855748 PMC4474297

[15] Günther A Krone O Svansson V Pohlmann A King J Hallgrimsson GT. Iceland as stepping stone for spread of highly pathogenic avian influenza virus between Europe and North America. Emerg Infect Dis. (2022) 28:2383–8. doi: 10.3201/eid2812.221086, PMID: 36261139 PMC9707596

[16] Webster RG Bean WJ Gorman OT Chambers TM Kawaoka Y. Evolution and ecology of influenza a viruses. Microbiol Rev. (1992) 56:152–79. doi: 10.1128/mr.56.1.152-179.1992, PMID: 1579108 PMC372859

[17] Roche B Lebarbenchon C Gauthier-Clerc M Chang C-M Thomas F Renaud F. Water-borne transmission drives avian influenza dynamics in wild birds: the case of the 2005–2006 epidemics in the Camargue area. Infect Genet Evol. (2009) 9:800–5. doi: 10.1016/j.meegid.2009.04.009, PMID: 19379841

[18] van den Brand JM Krone O Wolf PU van de Bildt MW van Amerongen G Osterhaus AD. Host-specific exposure and fatal neurologic disease in wild raptors from highly pathogenic avian influenza virus H5N1 during the 2006 outbreak in Germany. Vet Res. (2015) 46:24. doi: 10.1186/s13567-015-0148-5, PMID: 25879698 PMC4349770

[19] Nemeth NM Ruder MG Poulson RL Sargent R Breeding S Evans MN. Bald eagle mortality and Nest failure due to clade 2.3.4.4 highly pathogenic H5N1 influenza a virus. Sci Rep. (2023) 13:191. doi: 10.1038/s41598-023-27446-1, PMID: 36604450 PMC9813463

[20] Chitimia-Dobler L Schaper S Rieß R Bitterwolf K Frangoulidis D Bestehorn M. Imported Hyalomma ticks in Germany in 2018. Parasit Vectors. (2019) 12:134. doi: 10.1186/s13071-019-3380-4, PMID: 30909964 PMC6434826

[21] Cohen EB Auckland LD Marra PP Hamer SA. Avian migrants facilitate invasions of Neotropical ticks and tick-borne pathogens into the United States. Appl Environ Microbiol. (2015) 81:8366–78. doi: 10.1128/AEM.02656-15, PMID: 26431964 PMC4644638

[22] Mancuso E Toma L Pascucci I d’Alessio SG Marini V Quaglia M. Direct and indirect role of migratory birds in spreading CCHFV and WNV: a multidisciplinary study on three stop-over islands in Italy. Pathogens. (2022) 11:1056. doi: 10.3390/pathogens11091056, PMID: 36145488 PMC9505975

[23] Palomar AM Portillo A Santibáñez P Mazuelas D Arizaga J Crespo A. Crimean-Congo hemorrhagic fever virus in ticks from migratory birds, Morocco. Emerg Infect Dis. (2013) 19:260–3. doi: 10.3201/eid1902.121193, PMID: 23347801 PMC3559059

[24] Leblebicioglu H Eroglu C Erciyas-Yavuz K Hokelek M Acici M Yilmaz H. Role of migratory birds in spreading Crimean-Congo hemorrhagic fever. Turkey Emerg Infect Dis. (2014) 20:1331–4. doi: 10.3201/eid2008.131547, PMID: 25062428 PMC4111188

[25] Lindeborg M Barboutis C Ehrenborg C Fransson T Jaenson TGT Lindgren P-E. Migratory birds, ticks, and Crimean-Congo hemorrhagic fever virus. Emerg Infect Dis. (2012) 18:2095–7. doi: 10.3201/eid1812.120718, PMID: 23171591 PMC3557898

[26] Wilhelmsson P Jaenson TGT Olsen B Waldenström J Lindgren P-E. Migratory birds as disseminators of ticks and the tick-borne pathogens Borrelia Bacteria and tick-borne encephalitis (TBE) virus: a seasonal study at Ottenby bird Observatory in South-Eastern Sweden. Parasit Vectors. (2020) 13:607. doi: 10.1186/s13071-020-04493-5, PMID: 33272317 PMC7713317

[27] Wilhelmsson P Pawełczyk O Jaenson TGT Waldenström J Olsen B Forsberg P. Three Babesia species in *Ixodes ricinus* ticks from migratory birds in Sweden. Parasit Vectors. (2021) 14:183. doi: 10.1186/s13071-021-04684-8, PMID: 33794970 PMC8017608

[28] Wallner-Pendleton E. Hulet M. “Game Bird Industry,” in Foreign Animal Disease Preparedness and Response Plan (FAD PReP), editor. Poultry Industry Manual. Center for Food Security and Public Health; Iowa State University of Science and Technology: Ames, IA, USA (2013).

[29] Arroyo B Beja P. Impact of hunting management practices on biodiversity. Reconciling gamebird hunting and biodiversity (REGHAB) report. Brussels: European Commission Brussels (2002).

[30] Madden JR. How many gamebirds are released in the UK each year? Eur J Wildl Res. (2021) 67:72. doi: 10.1007/s10344-021-01508-z

[31] Aebischer NJ. Fifty-year trends in UK hunting bags of birds and mammals, and calibrated estimation of National bag Size, using GWCT’s National Gamebag Census. Eur J Wildl Res. (2019) 65:64. doi: 10.1007/s10344-019-1299-x

[32] Champagnon J Gauthier-Clerc M Lebreton J-D Mouronval J-B Guillemain M. Les Canards Colverts Lâchés Pour La Chasse Interagissent-Ils Avec Les Populations Sauvage. Faune Sauvage. (2013) 298:4–9.

[33] BirdLife International. Birds in Europe: Populations estimates, Trends and Conservation Status. Cambridge: BirdLife International (2004).

[34] U.S. Fish and Wildlife Service. Review of captive-reared mallard regulations on shooting preserves - final report. Washington DC: U.S. Fish and Wildlife Service (2013).

[35] Schummer ML Simpson J Shirkey B Kucia SR Lavretsky P Tozer DC. Population genetics and geographic origins of mallards harvested in northwestern Ohio. PLoS One. (2023) 18:e0282874. doi: 10.1371/journal.pone.0282874, PMID: 36920978 PMC10016643

[36] Lavretsky P McInerney NR Mohl JE Brown JI James HF McCracken KG. Assessing changes in genomic divergence following a century of human-mediated secondary contact among wild and captive-bred ducks. Mol Ecol. (2020) 29:578–95. doi: 10.1111/mec.15343, PMID: 31872482

[37] Miller RS Farnsworth ML Malmberg JL. Diseases at the livestock-wildlife interface: status, challenges, and opportunities in the United States. Prev Vet Med. (2013) 110:119–32. doi: 10.1016/j.prevetmed.2012.11.021, PMID: 23254245 PMC7127607

[38] Ayala AJ Yabsley MJ Hernandez SM. A review of pathogen transmission at the backyard chicken-wild bird Interface. Front Vet Sci. (2020) 7:7. doi: 10.3389/fvets.2020.539925, PMID: 33195512 PMC7541960

[39] Si Y de Boer WF Gong P. Different environmental drivers of highly pathogenic avian influenza H5N1 outbreaks in poultry and wild birds. PLoS One. (2013) 8:e53362. doi: 10.1371/journal.pone.0053362, PMID: 23308201 PMC3538778

[40] Vandegrift KJ Sokolow SH Daszak P Kilpatrick AM. Ecology of avian influenza viruses in a changing world. Ann N Y Acad Sci. (2010) 1195:113–28. doi: 10.1111/j.1749-6632.2010.05451.x, PMID: 20536820 PMC2981064

[41] Banyard AC Lean FZX Robinson C Howie F Tyler G Nisbet C. Detection of highly pathogenic avian influenza virus H5N1 clade 2.3.4.4b in great Skuas: a species of conservation concern in Great Britain. Viruses. (2022) 14:212. doi: 10.3390/v14020212, PMID: 35215806 PMC8878110

[42] Alexandrou O Malakou M Catsadorakis G. The impact of avian influenza 2022 on Dalmatian pelicans was the worst ever wildlife disaster in Greece. Oryx. (2022) 56:813–3. doi: 10.1017/S0030605322001041

[43] Gamarra-Toledo V Plaza PI Gutiérrez R Luyo P Hernani L Angulo F. Avian flu threatens Neotropical birds. Science. (1979) 379:246–6. doi: 10.1126/science.adg2271, PMID: 36656936

[44] Klaassen M Wille M. The plight and role of wild birds in the current bird flu Panzootic. Nat Ecol Evol. (2023) 7:1541–2. doi: 10.1038/s41559-023-02182-x, PMID: 37587226

[45] Stokstad E. Deadly flu spreads through north American birds. Science. (1979) 376:441–2. doi: 10.1126/science.abq7228, PMID: 35482856

[46] Hunter P. Europe’s worst ever bird flu outbreak. EMBO Rep. (2022) 23:e56048. doi: 10.15252/embr.202256048, PMID: 36102819 PMC9535796

[47] Abbasi J. Bird flu has begun to spread in mammals—Here’s What’s important to know. JAMA. (2023) 329:619–21. doi: 10.1001/jama.2023.1317, PMID: 36753673

[48] Ganar K Das M Sinha S Kumar S. Newcastle disease virus: current status and our understanding. Virus Res. (2014) 184:71–81. doi: 10.1016/j.virusres.2014.02.016, PMID: 24589707 PMC7127793

[49] Molaei G Thomas MC Muller T Medlock J Shepard JJ Armstrong PM. Dynamics of vector-host interactions in avian communities in four eastern equine encephalitis virus foci in the northeastern U.S. PLoS Negl Trop Dis. (2016) 10:e0004347. doi: 10.1371/journal.pntd.0004347 26751704 PMC4713425

[50] Dhama K Saminathan M Karthik K Tiwari R Shabbir MZ Kumar N. Avian rotavirus enteritis – an updated review. Vet Q. (2015) 35:142–58. doi: 10.1080/01652176.2015.1046014, PMID: 25917772

[51] Armstrong PM Andreadis TG. Eastern equine encephalitis virus — old enemy, new threat. N Engl J Med. (2013) 368:1670–3. doi: 10.1056/NEJMp1213696, PMID: 23635048

[52] Parashar UD Burton A Lanata C Boschi-Pinto C Shibuya K Steele D. Global mortality associated with rotavirus disease among children in 2004. J Infect Dis. (2009) 200:S9–S15. doi: 10.1086/605025, PMID: 19817620

[53] Chen S. Ultrafast one-pass FASTQ data preprocessing, quality control, and deduplication using Fastp. iMeta. (2023) 2:e107. doi: 10.1002/imt2.107 PMC10989850 38868435

[54] Chen S Zhou Y Chen Y Gu J. Fastp: an ultra-fast all-in-one FASTQ preprocessor. Bioinformatics. (2018) 34:i884–90. doi: 10.1093/bioinformatics/bty560, PMID: 30423086 PMC6129281

[55] Li D Liu C-M Luo R Sadakane K Lam T-W. MEGAHIT: an ultra-fast single-node solution for large and complex metagenomics assembly via succinct de Bruijn graph. Bioinformatics. (2015) 31:1674–6. doi: 10.1093/bioinformatics/btv033, PMID: 25609793

[56] Buchfink B Reuter K Drost H-G. Sensitive protein alignments at tree-of-life scale using DIAMOND. Nat Methods. (2021) 18:366–8. doi: 10.1038/s41592-021-01101-x, PMID: 33828273 PMC8026399

[57] Buchfink B Xie C Huson DH. Fast and Sensitive Protein Alignment Using DIAMOND. Nat Methods. (2015) 12:59–60. doi: 10.1038/nmeth.3176, PMID: 25402007

[58] Bağcı C Patz S Huson DH. DIAMOND+MEGAN: fast and easy taxonomic and functional analysis of short and long microbiome sequences. Curr Protoc. (2021) 1:e59. doi: 10.1002/cpz1.59 33656283

[59] Huson DH Albrecht B Bağcı C Bessarab I Górska A Jolic D. MEGAN-LR: new algorithms allow accurate binning and easy interactive exploration of metagenomic long reads and Contigs. Biol Direct. (2018) 13:6. doi: 10.1186/s13062-018-0208-7, PMID: 29678199 PMC5910613

[60] Gouy M Guindon S Gascuel O. SeaView version 4: a multiplatform graphical user Interface for sequence alignment and phylogenetic tree building. Mol Biol Evol. (2010) 27:221–4. doi: 10.1093/molbev/msp259, PMID: 19854763

[61] Capella-Gutiérrez S Silla-Martínez JM Gabaldón T. TrimAl: a tool for automated alignment trimming in large-scale phylogenetic analyses. Bioinformatics. (2009) 25:1972–3. doi: 10.1093/bioinformatics/btp348, PMID: 19505945 PMC2712344

[62] Minh BQ Schmidt HA Chernomor O Schrempf D Woodhams MD von Haeseler A. IQ-TREE 2: new models and efficient methods for phylogenetic inference in the genomic era. Mol Biol Evol. (2020) 37:1530–4. doi: 10.1093/molbev/msaa015, PMID: 32011700 PMC7182206

[63] Martin DP Murrell B Khoosal A Muhire B. Detecting and analyzing genetic recombination using RDP4 (2017) 1525:433–60. doi: 10.1007/978-1-4939-6622-6_17, 27896731

[64] Collier AM Lyytinen OL Guo YR Toh Y Poranen MM Tao YJ. Initiation of RNA polymerization and polymerase Encapsidation by a small DsRNA virus. PLoS Pathog. (2016) 12:e1005523. doi: 10.1371/journal.ppat.1005523, PMID: 27078841 PMC4831847

[65] Delmas B Attoui H Ghosh S Malik YS Mundt E Vakharia VN. ICTV Virus Taxonomy Profile: Picobirnaviridae. J Gen Virol. (2019) 100:133–4. doi: 10.1099/jgv.0.001186, PMID: 30484763 PMC12662030

[66] Wang F Wang M Dong Y Zhang B Zhang D. Genetic characterization of a novel Calicivirus from a goose. Arch Virol. (2017) 162:2115–8. doi: 10.1007/s00705-017-3302-8 28289976

[67] Wang X Liu N Wang F Ning K Li Y Zhang D. Genetic characterization of a novel duck-origin picornavirus with six 2A proteins. J Gen Virol. (2014) 95:1289–96. doi: 10.1099/vir.0.063313-0, PMID: 24659102

[68] Mitchell DB Both GW. Conservation of a potential metal binding motif despite extensive sequence diversity in the rotavirus nonstructural protein NS53. Virology. (1990) 174:618–21. doi: 10.1016/0042-6822(90)90117-a, PMID: 2154894

[69] Chen F Knutson TP Porter RE Ciarlet M Mor SK Marthaler DG. Genome characterization of Turkey rotavirus G strains from the United States identifies potential recombination events with human rotavirus B strains. J Gen Virol. (2017) 98:2931–6. doi: 10.1099/jgv.0.000963, PMID: 29168675 PMC5845698

[70] Huang P Sun L Li J Wu Q Rezaei N Jiang S. Potential cross-species transmission of highly pathogenic avian influenza H5 subtype (HPAI H5) viruses to humans calls for the development of H5-specific and universal influenza vaccines. Cell Discov. (2023) 9:58. doi: 10.1038/s41421-023-00571-x, PMID: 37328456 PMC10275984

[71] Drilling N Titman RD McKinney F. Mallard (*Anas Platyrhynchos*) In: Billerman SM, editor. Birds of the world. Ithaca, NY: Cornell Lab of Ornithology (2020)

[72] Vibin J Chamings A Klaassen M Alexandersen S. Metagenomic characterisation of additional and novel avian viruses from Australian wild ducks. Sci Rep. (2020) 10:22284. doi: 10.1038/s41598-020-79413-9, PMID: 33335272 PMC7747739

[73] Ebnalnassir M Ghalyanchi Langeroudi A Najafi H Hosseini H Ziafati Kafi Z Sadri N. Molecular identification and phylogenetic analysis of a novel strain of duck picornavirus (Aalivirus) in Iran: the first report. Iran J Virol. (2022) 16:54–8.

[74] Yugo DM Hauck R Shivaprasad HL Meng X-J. Hepatitis virus infections in poultry. Avian Dis. (2016) 60:576–88. doi: 10.1637/11229-070515-Review.1 27610716

[75] Vibin J Chamings A Klaassen M Bhatta TR Alexandersen S. Metagenomic characterisation of avian parvoviruses and picornaviruses from Australian wild ducks. Sci Rep. (2020) 10:12800. doi: 10.1038/s41598-020-69557-z, PMID: 32733035 PMC7393117

[76] Abrantes J van der Loo W Le Pendu J Esteves PJ. Rabbit Haemorrhagic disease (RHD) and rabbit Haemorrhagic disease virus (RHDV): a review. Vet Res. (2012) 43:12. doi: 10.1186/1297-9716-43-12, PMID: 22325049 PMC3331820

[77] Hofmann-Lehmann R Hosie MJ Hartmann K Egberink H Truyen U Tasker S. Calicivirus infection in cats. Viruses. (2022) 14:937. doi: 10.3390/v14050937, PMID: 35632680 PMC9145992

[78] Guo M Evermann JF Saif LJ. Detection and molecular characterization of cultivable Caliciviruses from clinically Normal mink and enteric Caliciviruses associated with diarrhea in mink. Arch Virol. (2001) 146:479–93. doi: 10.1007/s007050170157, PMID: 11338385 PMC7086843

[79] Ike AC Roth BN Böhm R Pfitzner AJP Marschang RE. Identification of bovine enteric Caliciviruses (BEC) from cattle in Baden-Württemberg. Dtsch Tierarztl Wochenschr. (2007) 114:12–5. 17252930

[80] Katpally U Smith TJ. The Caliciviruses. Curr Top Microbiol Immunol. (2010) 343:23–41. doi: 10.1007/82_2010_36 20376611

[81] Goodgame RW. Viral causes of diarrhea. Gastroenterol Clin N Am. (2001) 30:779–95. doi: 10.1016/s0889-8553(05)70210-7, PMID: 11586557

[82] Hall AJ Lopman BA Payne DC Patel MM Gastañaduy PA Vinjé J. Norovirus disease in the United States. Emerg Infect Dis. (2013) 19:1198–205. doi: 10.3201/eid1908.130465, PMID: 23876403 PMC3739528

[83] Karst SM. Pathogenesis of noroviruses, emerging RNA viruses. Viruses. (2010) 2:748–81. doi: 10.3390/v2030748, PMID: 21994656 PMC3185648

[84] Canuti M Kroyer ANK Ojkic D Whitney HG Robertson GJ Lang AS. Discovery and characterization of novel RNA viruses in aquatic north American wild birds. Viruses. (2019) 11:768. doi: 10.3390/v11090768, PMID: 31438486 PMC6784231

[85] Canuti M Wilson L Bowes V Redford T Dufour SC Lang AS. A novel Calicivirus discovered in trumpeter swans (Cygnus Buccinator) expands the richness of known avian Caliciviruses. Curr Res Microb Sci. (2022) 3:100169. doi: 10.1016/j.crmicr.2022.100169, PMID: 36518169 PMC9742997

[86] Day JM Ballard LL Duke MV Scheffler BE Zsak L. Metagenomic analysis of the Turkey gut RNA virus community. Virol J. (2010) 7:313. doi: 10.1186/1743-422X-7-313, PMID: 21073719 PMC2991317

[87] Reddy MV Gupta V Nayak A Tiwari SP. Picobirnaviruses in animals: a review. Mol Biol Rep. (2023) 50:1785–97. doi: 10.1007/s11033-022-08133-2, PMID: 36462086 PMC9734933

[88] Wang Y Bányai K Tu X Jiang B. Simian Genogroup I Picobirnaviruses: prevalence, genetic diversity, and zoonotic potential. J Clin Microbiol. (2012) 50:2779–82. doi: 10.1128/JCM.00634-12, PMID: 22622441 PMC3421516

[89] Bányai K Martella V Bogdán Á Forgách P Jakab F Meleg E. Genogroup I Picobirnaviruses in pigs: evidence for genetic diversity and relatedness to human strains. J Gen Virol. (2008) 89:534–9. doi: 10.1099/vir.0.83134-0, PMID: 18198385

[90] Li W Qiang X Qin S Huang Y Hu Y Bai B. Virome diversity analysis reveals novel enteroviruses and a human Picobirnavirus in stool samples from African green monkeys with diarrhea. Infect Genet Evol. (2020) 82:104279. doi: 10.1016/j.meegid.2020.104279, PMID: 32165243 PMC7102571

[91] Ramesh A Bailey ES Ahyong V Langelier C Phelps M Neff N. Metagenomic characterization of swine slurry in a north American swine farm operation. Sci Rep. (2021) 11:16994. doi: 10.1038/s41598-021-95804-y, PMID: 34417469 PMC8379149

[92] Parashar UD Bresee JS Gentsch JR Glass RI. Rotavirus. Emerg Infect Dis. (1998) 4:561–70. doi: 10.3201/eid0404.980406, PMID: 9866732 PMC2640254

[93] Trojnar E Otto P Roth B Reetz J Johne R. The genome segments of a group D rotavirus possess group A-like conserved termini but encode group-specific proteins. J Virol. (2010) 84:10254–65. doi: 10.1128/JVI.00332-10, PMID: 20631147 PMC2937790

[94] Johne R Otto P Roth B Löhren U Belnap D Reetz J. Sequence analysis of the VP6-encoding genome segment of avian group F and G rotaviruses. Virology. (2011) 412:384–91. doi: 10.1016/j.virol.2011.01.031, PMID: 21329955

[95] Otto PH Ahmed MU Hotzel H Machnowska P Reetz J Roth B. Detection of avian rotaviruses of groups a, D, F and G in diseased chickens and turkeys from Europe and Bangladesh. Vet Microbiol. (2012) 156:8–15. doi: 10.1016/j.vetmic.2011.10.001, PMID: 22079218 PMC7117391

[96] Gerba CP Rose JB Haas CN Crabtree KD. Waterborne rotavirus: a risk assessment. Water Res. (1996) 30:2929–40. doi: 10.1016/S0043-1354(96)00187-X

[97] Cook N Bridger J Kendall K Gomara MI El-Attar L Gray J. The zoonotic potential of rotavirus. J Infect. (2004) 48:289–302. doi: 10.1016/j.jinf.2004.01.018 15066329

[98] Geletu US Usmael MA Bari FD. Rotavirus in calves and its zoonotic importance. Vet Med Int. (2021) 2021:6639701–18. doi: 10.1155/2021/6639701, PMID: 33968359 PMC8081619

[99] McDonald SM Nelson MI Turner PE Patton JT. Reassortment in segmented RNA viruses: mechanisms and outcomes. Nat Rev Microbiol. (2016) 14:448–60. doi: 10.1038/nrmicro.2016.46, PMID: 27211789 PMC5119462

[100] Fujii Y Hirayama M Nishiyama S Takahashi T Okajima M Izumi F. Characterization of an avian rotavirus a strain isolated from a velvet scoter (Melanitta Fusca): implication for the role of migratory birds in global spread of avian rotaviruses. J Gen Virol. (2022) 103:103 (2). doi: 10.1099/jgv.0.001722, PMID: 35175915

[101] Pepin KM Leach CB Barrett NL Ellis JW VanDalen KK Webb CT. Environmental transmission of influenza a virus in mallards. MBio. (2023) 14:e0086223. doi: 10.1128/mbio.00862-23, PMID: 37768062 PMC10653830

[102] Hofer U. Multitude of viruses in game animals. Nat Rev Microbiol. (2022) 20:253–3. doi: 10.1038/s41579-022-00716-x, PMID: 35236931 PMC8889867

[103] Dwight IA Coates PS Stoute ST Pitesky ME. Health surveillance of a potential bridge host: pathogen exposure risks posed to avian populations augmented with captive-bred pheasants. Transbound Emerg Dis. (2022) 69:1095–107. doi: 10.1111/tbed.14068, PMID: 33711203 PMC9290078

[104] Davis JB Outlaw DC Ringelman KM Kaminski RM Lavretsky P. Low levels of hybridization between domestic and wild mallards wintering in the lower Mississippi flyway. Ornithology. (2022) 139:139 (4). doi: 10.1093/ornithology/ukac034

[105] Hou Z-C Yang F-X Qu L-J Zheng J-X Brun J-M Basso B. Genetic structure of Eurasian and north American mallard ducks based on MtDNA data. Anim Genet. (2012) 43:352–5. doi: 10.1111/j.1365-2052.2011.02248.x, PMID: 22486512

[106] Drovetski SV. Survey of potential viral pathogens in the ceca of pen-raised mallards. U.S. Geological Survey data release, (2024) doi: 10.5066/P9FLGWOB

