# Supplementary material for: Viral pathogen detection in U.S. game-farm mallard (Anas platyrhynchos) flags spillover risk to wild birds
Source: Front Vet Sci. 2024 May 27;11:1396552. doi: 10.3389/fvets.2024.1396552 (PMC11163284; doi:10.3389/fvets.2024.1396552)
Supplement: Supplementary file 1 [file Image_1.pdf]

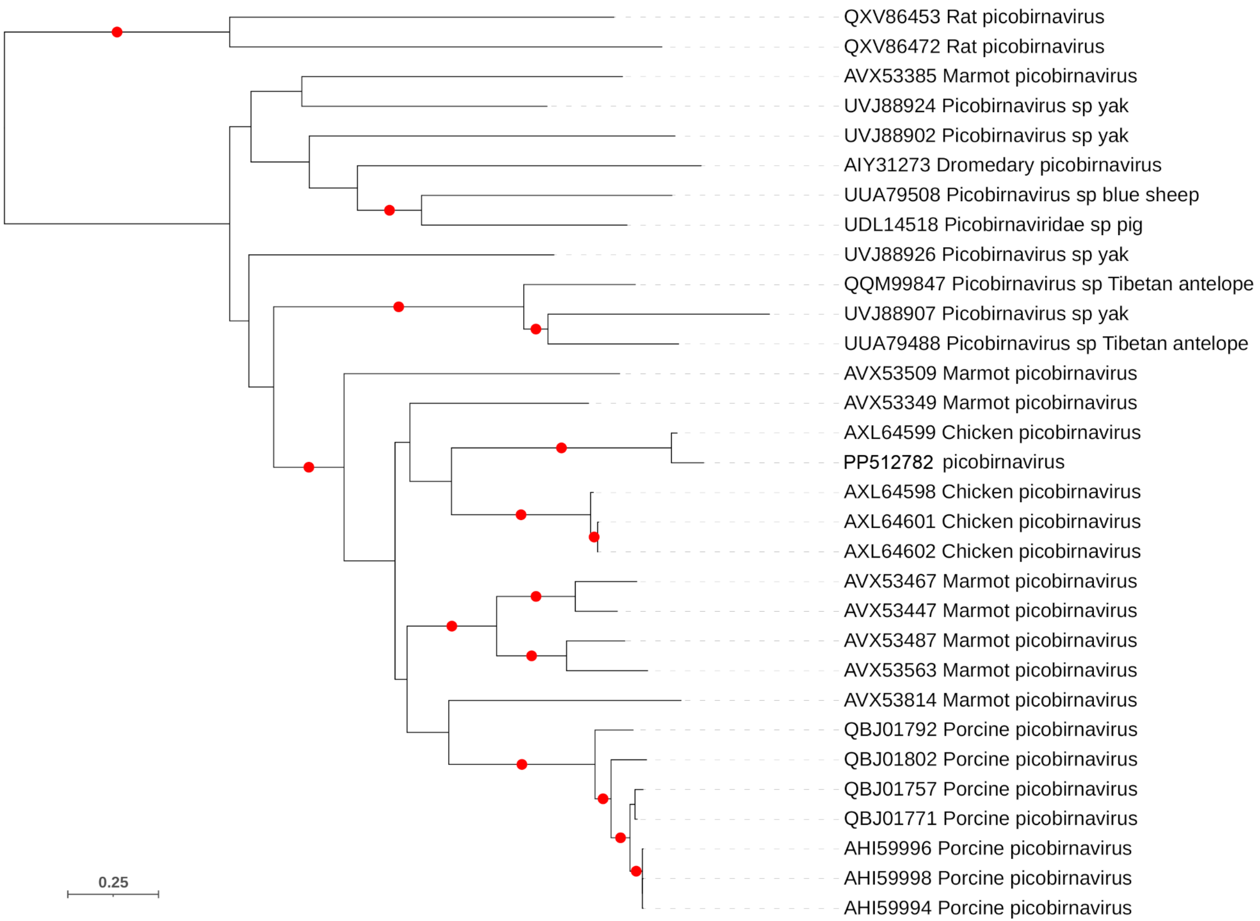

Supplementary figure 1. Maximum likelihood (extended majority-rule consensus) tree of the family Picobirnaviridae using the ORF3 region. Red circles indicate branches with UFBoot support greater than 95%.
