# Supplementary material for: Viral pathogen detection in U.S. game-farm mallard (Anas platyrhynchos) flags spillover risk to wild birds
Source: Front Vet Sci. 2024 May 27;11:1396552. doi: 10.3389/fvets.2024.1396552 (PMC11163284; doi:10.3389/fvets.2024.1396552)
Supplement: Supplementary file 3 [file Image_3.pdf]

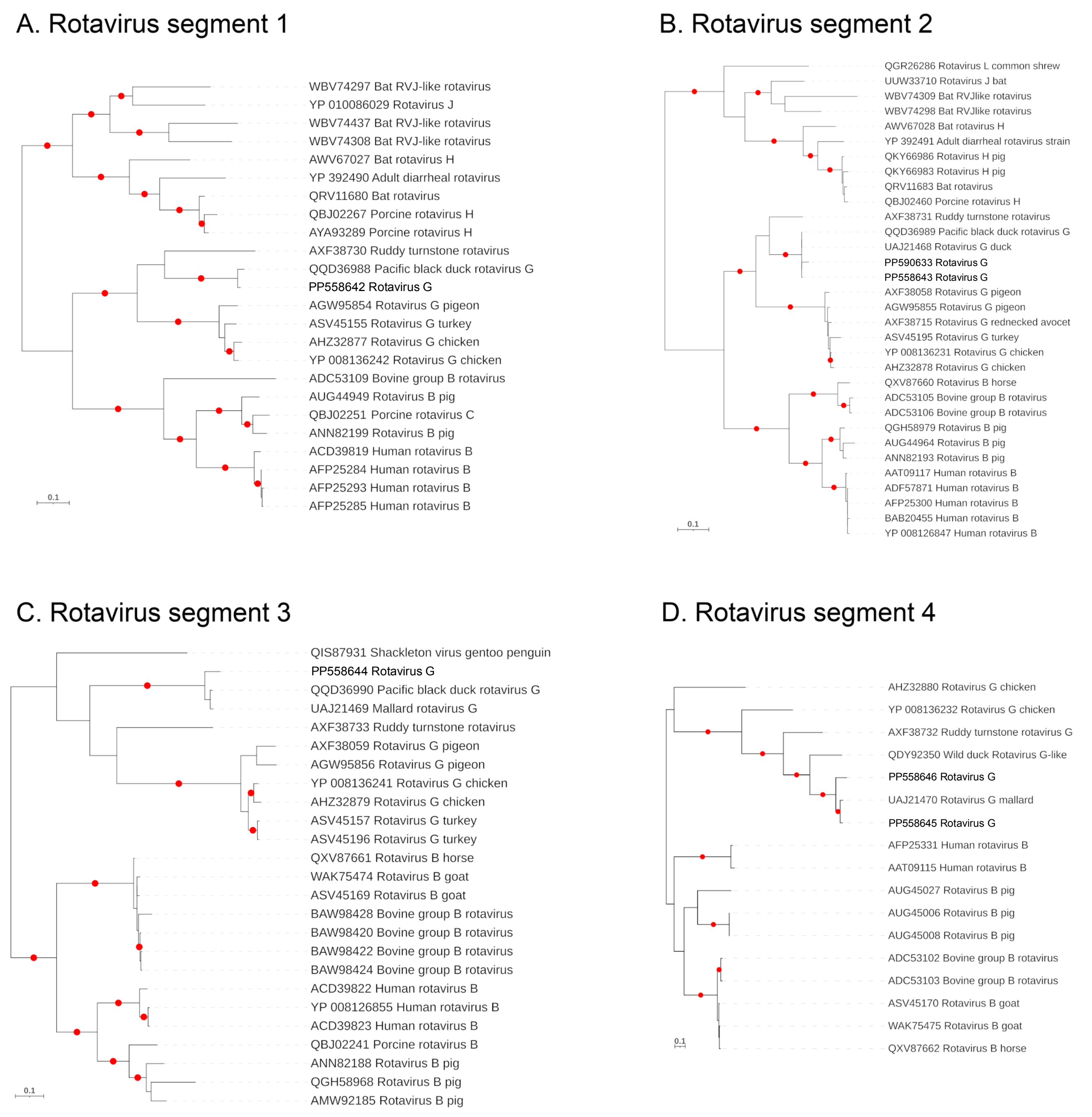

Supplementary figure 3. Maximum likelihood (extended majority-rule consensus) tree of Rotavirus G and its closest relatives from Segment 1 (A), Segment 2 (B), Segment 3 (C), and Segment 4 (D). Red circles indicate branches with UFBoot support greater than 95%.
